# Book Notices

**Published:** 1855-12

**Authors:** 


THE Profession will be gratified to know that Prof. Brainard of this city, contemplates publishing a volume which shall embody his own Surgical experience and improvements, together with reports of numerous illustrative cases. It is not designed to be a text book on surgery, but one which will meet the wants of the profession by supplying what in most of our systematic works is entirely wanting or but partially developed.

The miscroscopic elements of the blood and tissues in certain diseased conditions will occupy a portion of its contents and the whole will be illustrated with drawings from nature.

It will be issued from the press during the coming year. J.

